# A Design of Experiment Approach to Optimize Spray-Dried Powders Containing *Pseudomonas aeruginosa* *Podoviridae* and *Myoviridae* Bacteriophages

**DOI:** 10.3390/v13101926

**Published:** 2021-09-24

**Authors:** Emilie Tabare, Tea Glonti, Christel Cochez, Cyrille Ngassam, Jean-Paul Pirnay, Karim Amighi, Jonathan Goole

**Affiliations:** 1 Laboratory of Pharmaceutics and Biopharmaceutics, Faculty of Pharmacy, ULB, 1050 Brussels, Belgium; karim.amighi@ulb.be (K.A.); jonathan.goole@ulb.be (J.G.); 2 Laboratory for Molecular and Cellular Technology, Queen Astrid Military Hospital, 1120 Brussels, Belgium; tea.glonti@mil.be (T.G.); christel.cochez@mil.be (C.C.); jean-paul.pirnay@mil.be (J.-P.P.); 3 Vesale Pharma S.A., 5310 Noville-sur-Mehaigne, Belgium; c.ngassam@vesalepharma.com

**Keywords:** DoE, QbD, antibiotic-resistant, bacteriophage therapy, spray-drying, drying, stability, *Pseudomonas aeruginosa*, LUZ19, 14-1

## Abstract

In the present study, we evaluated the effect of spray-drying formulations and operating parameters of a laboratory-scale spray-dryer on the characteristics of spray-dried powders containing two *Pseudomonas aeruginosa* bacteriophages exhibiting different morphotypes: a podovirus (LUZ19) and a myovirus (14-1). We optimized the production process for bacteriophage-loaded powders, with an emphasis on long-term storage under ICH (international conference on harmonization) conditions. D-trehalose-/L-isoleucine-containing bacteriophage mixtures were spray-dried from aqueous solutions using a Büchi Mini Spray-dryer B-290 (Flawil, Switzerland). A response surface methodology was used for the optimization of the spray-drying process, with the following as-evaluated parameters: Inlet temperature, spray gas flow rate, and the D-trehalose/L-isoleucine ratio. The dried powders were characterized in terms of yield, residual moisture content, and bacteriophage lytic activity. L-isoleucine has demonstrated a positive impact on the activity of LUZ19, but a negative impact on 14-1. We observed a negligible impact of the inlet temperature and a positive correlation of the spray gas flow rate with bacteriophage activity. After optimization, we were able to obtain dry powder preparations of both bacteriophages, which were stable for a minimum of one year under different ICH storage conditions (up to and including 40 °C and 75% relative humidity).

## 1. Introduction

*Pseudomonas aeruginosa* is a Gram-negative bacterium responsible for a variety of infections, ranging from mild skin infections to life-threatening systemic infections, which are difficult to treat due to the bacterium’s intrinsic and acquired resistance mechanisms to a wide range of antibiotics [1]. The application of lytic bacteriophages (phages) is one of the possible solutions to counter such resistance. They have been used since the 1920s to treat bacterial infections but have been neglected in Western medicine in favor of antibiotics. Phage therapy has now been rekindled as an additional tool in the fight against multidrug-resistant bacteria [2].

To date, bacteriophages have often been administered as suspensions, which are stable for short (weeks) to medium (months) storage periods at 2–8 °C. Sometimes, stabilizing agents (e.g., albumins, salts, or gelatin) are added, in order to enhance bacteriophage stability [3]. Freezing (e.g., at −80 °C) or freeze-drying are used for long-term storage purposes [4]; however, both methods are relatively complex, expensive, and energy-consuming.

Spray-drying has been widely used in the pharmaceutical industry. As a fast, continuous, reproducible, and single-step method, it could advantageously replace freeze-drying as a means for the long-term preservation of bacteriophages in a solid state. Moreover, spray-drying can be used as a particle engineering method to produce stabilized dried particles with various forms, sizes, and crystallinity, compatible with various routes of administration [5].

The spray-drying process has already been used to dry bacteriophages; in particular, for pulmonary administration. In 2011, Matinkhoo et al. spray-dried bacteriophages KS4-M, KS14, and cocktails of bacteriophages ΦKZ/D3 and ΦKZ/D3/KS4-M using a Büchi Nano Spray-Dryer B-90 (Büchi Labortechnik AG, Flawil, Switzerland) equipped with a vibrating mesh atomizer [6]. KS4-M and KS14 are *Burkholderia cepacia* bacteriophages with *Myoviridae* morphology, while ΦKZ and D3 are *P. aeruginosa* bacteriophages exhibiting *Myoviridae* and *Siphoviridae* morphologies, respectively. A relatively low feed flow rate of 0.33 mL/min was applied. All of the bacteriophages were successfully dried, with an overall titer loss lower than 1 log_10_ plaque-forming units (pfu) per mL. An elaborate storage stability study could not be performed, as the produced batch sizes were too small. Indeed, only one titer retest (in triplicate), after three months of refrigerated storage of bacteriophage ΦKZ/D3 cocktail in leucine/trehalose and trehalose/leucine/casein formulations was carried out, showing less than 0.15 log_10_ pfu/mL titer loss. However, this study demonstrated that it was feasible to spray-dry bacteriophages while maintaining an acceptable lytic activity (i.e., a loss of activity lower than 1 log_10_ pfu/mL) after storage in a refrigerator for three months. Nevertheless, long-term storage stability studies should be performed at different temperatures and relative humidities.

In 2013, Vandenheuvel et al. obtained a reduction of lytic activity of less than 1 log_10_ pfu/mL for the *P. aeruginosa* bacteriophage LUZ19 (podoviridus), but more than 2.5 log_10_ pfu/mL for the *Staphylococcus aureus* bacteriophage Romulus (myoviridus) after spray-drying [7]. They hypothesized that an elevated atomizing airflow had caused an increased reduction of bacteriophage titer and that the deleterious shear forces could be reduced by applying a gentler atomizing technique using an ultrasonic nozzle. In 2014, they assessed the storage stability of spray-dried powder samples of trehalose containing mixtures of bacteriophages LUZ19 and Romulus under four different storage conditions: 4 °C and 0% relative humidity (RH), 4 °C and 54% RH, 25 °C and 0% RH, and 25 °C and 54% RH [8]. For LUZ19, the activity was shown to remain stable at 4 °C and 0% RH for at least one year while, at 25 °C, a loss of 3 log_10_ pfu/mL was observed after 7 months. For Romulus, a loss of approximately 2 log_10_ pfu/mL was observed after three months at 4 °C and more than 4 log_10_ pfu/mL at 25 °C, independent of the RH. After investigation, they concluded that trehalose-based particles required specific storage conditions to limit crystallization issues. To prevent crystallization, it was shown that the RH must be properly controlled. In addition, they observed that the storage temperature greatly influenced the viability of the bacteriophage. Over time, the bacteriophage titer declined at 25 °C, even when no crystallization of the amorphous trehalose matrix was observed. In contrast, it remained stable at 4 °C. The effects of crystal formation on bacteriophage viability appeared to be bacteriophage-specific. They concluded that larger bacteriophage virions were more prone to inactivation upon crystal formation than smaller bacteriophage virions.

In 2016, Leung et al. spray-dried *P. aeruginosa* bacteriophage PEV2 (podovirus) with trehalose, mannitol, and L-leucine. A significant titer loss (~2 log_10_ pfu/mL) was noted when using an ultrasonic nozzle employed in a spray-freeze-drying approach. However, the conventional two-fluid nozzle used in the spray-drying method was less detrimental (~0.75 log_10_ loss), in contradiction with the hypotheses previously proposed by Vandenheuvel et al. [9].

In 2017, Leung et al. conducted a study to evaluate bacteriophage viability in spray-dried inhalable powders after storage at 4 °C and 0%, and 22% and 60% RH during 12 months [10]. All powders were partially crystalline, but significant crystallization issues appeared when the powders were stored at an RH higher than 22%. Moreover, it was demonstrated that bacteriophages did not survive one year of storage at 60% RH. In 2018, the same research group conducted another experiment to study the influence of leucine content and storage temperature on the long-term stability of spray-dried trehalose powders containing two different bacteriophages (podovirus PEV2 and myovirus PEV40) [11]. For both, a low activity loss after spray-drying was observed (0.7–0.8 log_10_ pfu/mL and 0.2–0.3 log_10_ pfu/mL, respectively). Moreover, after storage at 4 °C PEV2, bacteriophage powders were stable for both studied formulations (70:30 and 60:40% *w*/*w* trehalose and L-leucine, respectively). At 20 °C, the formulation with a lower amount of leucine (30% *w*/*w*) preserved the viability of PEV2 bacteriophages but, for the formulation with 40% *w*/*w*, ~0.9 log_10_ pfu/mL storage loss was observed after one year. Regarding PEV40, bacteriophage powders were characterized by lower stability during storage. A 0.5 log_10_ pfu/mL loss was noted for both formulations and storage temperatures after one year. For storage, the produced powders were aliquoted into scintillation vials and packed inside a vacuum-sealed bag using a Westinghouse vacuum food sealer, and kept inside an RH-controlled chamber with an RH lower than 20% [11].

In 2018, Chang et al. used a Taguchi experimental design with a funneling approach to identify the most appropriate excipients to protect PEV1, PEV20, and PEV61 bacteriophages during spray-drying. They concluded that formulations containing a mixture of lactose or trehalose with leucine were efficient in limiting the loss of activity of the dried bacteriophages to less than 1 log_10_ pfu/mL [12]. In 2019, they demonstrated that spray-dried bacteriophage PEV (PEV1, PEV20, and PEV61) powders containing lactose and leucine were biologically and physically stable over long-term storage periods (up to 12 months) at ambient temperature and 60% RH. The bacteriophage powders were heat-sealed in aluminum pouches inside an acrylic box maintained at 15% RH [13]. It would have been interesting if they had performed this experiment in opened containers in order to allow for an evaluation of the stability of the bacteriophages at 60% RH.

In 2020, Carrigy et al. developed a stable anti-campylobacter bacteriophage (CP30A) powder with 0.6 log_10_ pfu/mL loss of titer after formulation, spray-drying, and one month of storage at room temperature. This result was obtained through process modeling, supplement phase diagram, and micro particle engineering [14]. Unfortunately, they did not report results beyond one month of storage.

Chang et al. conducted a study to investigate the stabilization mechanism of bacteriophages that were contained in pharmaceutical solids [15]. PEV20 (myovirus) bacteriophages were co-spray-dried with lactose and leucine to generate partially crystalline particles. They found that bacteriophage viability was closely associated with the temperature gap between the storage temperature (Ts) and the glass transition temperature (Tg) of bacteriophage-loaded powders containing lactose and leucine. The bacteriophages remained stable when the (Tg – Ts) value was above 46 °C. Similar effects were observed for powders containing 50% or 80% *w*/*w* of lactose. These findings corroborated the vitrification hypothesis for bacteriophage stabilization.

To the best of our knowledge, there was no report on the optimal design of experiment approach to spray-dry phage and stability study of bacteriophage loaded spray-dried powder at 4 ± 2 °C or 25 ± 2 °C and 60% RH; 30 ± 2 °C and 65% RH; and 40 ± 2 °C and 75% RH. In the present study, our primary objective was to better understand the influence on bacteriophage activity of certain spray-dryer parameters, which are reported to be deleterious (drying temperature in the column and spray-gas flow) as well as L-isoleucine concentration. A response surface randomized I-optimal design was devised to assess the effect of the spray-drying process parameters and formulations on powder process yield, residual moisture content (RMC), process outlet temperature (which is the temperature of the drying gas at the end of the column; Tout), and loss of bacteriophage lytic activity (expressed as log10 pfu/mg).

## 2. Materials and Methods

### 2.1. Materials

**Figure 1:**
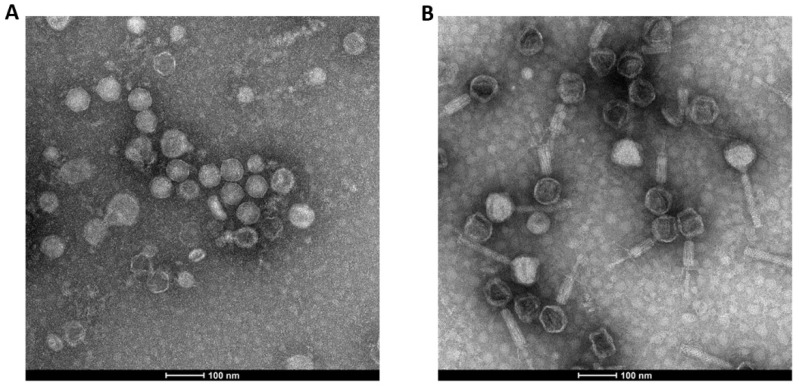
Transmission electron microscopy (TEM) pictures of LUZ19 (**A**) and 14-1 (**B**) using a Tecnai Spirit microscope (FEI, Eindhoven, The Netherlands) operating at 120 kV, with a spot size of 1. Scale bar is 100 nm.

Two lytic *P. aeruginosa* bacteriophages of different morphologies, a podovirus (LUZ19; Figure 1A) and a myovirus (14-1; Figure 1B), both with propagation strain PAO1K (2.0 × 10^11^ pfu/mL stock titer), were used as models. Bacteriophage LUZ19 [16] was provided by Professor Rob Lavigne (KU Leuven, Belgium). Bacteriophage 14-1 and propagation strain PAO1K were obtained from the Queen Astrid Military Hospital (Brussels, Belgium). LUZ19 is characterized by a non-enveloped head with icosahedral symmetry and a diameter of approximately 75 nm (length) and 65 nm (width). Bacteriophage 14-1 has a non-enveloped head with icosahedral symmetry and a diameter of approximately 80 nm, a tubular tail with helical symmetry, 20–25 nm in diameter, and 150 nm long in its non-contracted state. The tail consists of a central tube, a contractile sheath, a collar, a base plate, and terminal fibers.

D-(+)-trehalose dihydrate and L-isoleucine of non-animal origin were used as excipients, which were purchased from Sigma-Aldrich (Overijse, Brussel). Milli-Q (ELGA) water (>18.2 MΩ) was used as a solvent.

### 2.2. Methods

#### 2.2.1. Spray-Drying

Formulations containing different D-(+)-trehalose dihydrate and L-isoleucine weight ratios (namely, 60:40, 70:30, 80:20, and 90:10% *w*/*w*) were spray-dried as 5% (*w*/*v*) aqueous solutions with 1% *v*/*v* of both bacteriophages separately using a Büchi Mini Spray-dryer B-290 (Flawil, Switzerland) with a Büchi dehumidifier B296 (Flawil, Switzerland). The experiments were performed in the open mode, coupled with a 0.7 mm two-fluid nozzle and using air as the drying gas in a co-current mode. The suspension was fed at a constant feed rate of 2 mL/min and an aspiration rate of 35 m^3^/h. T_in_ (temperature of the drying gas at the top of the column), spray gas flow, and L-isoleucine concentration were set according to the requirements of the individual runs, as determined in the design study (Section 2.2.11). After processing, each powder was stored in sealed type-1 clear pharmaceutical grade glass amber vials under nitrogen at different ICH (International Council for Harmonization of Technical Requirements for Pharmaceuticals for Human Use) conditions. The stoppers used were ultra-pure chlorobutyl-isoprene rubber stoppers, sealed to the vial with aluminum caps.

#### 2.2.2. Production Yield

Yields were calculated based on the weight of powder that was collected after spray-drying in the spray dryer collection vessel and are expressed as a percentage of the weight of dry materials introduced into the feed solution (*w*/*w*%).

#### 2.2.3. Scanning Electron Microscopy

Scanning Electron Microscopy (SEM), using an SU8020 SEM-FEG microscope (Hitachi, Chiyoda, Tokyo, Japan), was performed to evaluate the morphology of the dried particles. The samples were prepared by sticking the powder to a circular shelf using an adhesive carbon tab before coating with 2.5 nm of Pt/Pd using a Leica ACE600 metallizer. The images were acquired at a voltage of 5 kV using the secondary electron detector SE (L) located in the chamber. The higher magnification images were acquired at a voltage of 2 kV, using the SE (U) detector located in the column. The magnifications used were 1000, 5000, 30,000, and 50,000×.

#### 2.2.4. Thermal Analysis

The amount of residual water was assessed by thermogravimetric analysis (TGA), using a TA instruments Q500 apparatus and Universal Analysis 2000 (version 4.4A) software (TA Instruments, New Castle, DL, USA). About 10 mg of powder were placed on the platinum cup and were subjected to a range of temperatures from 30 to 200 °C with a heating rate of 10 °C/min. The remaining moisture content (RMC) was defined as the weight loss between the powders at 30 °C and 120 °C.

Differential scanning calorimetry (DSC) analysis was carried out under nitrogen purge using a DSC Q2000 instrument and Universal Analysis 2000 (version 4.4A) software (TA Instruments, Asse, Belgium), in order to determine Tg. Powder samples (2–4 mg) were placed in a Tzero hermetic aluminum pan, sealed, and heated over the temperature range of −50 to 150 °C with a heating rate of 10 °C/min.

#### 2.2.5. Dynamic Vapor Sorption

The sorption–desorption isotherms of the samples were determined using the dynamic vapor sorption (DVS) technique. The DVS experiments were conducted using a Q5000 SA instrument (TA instruments, Asse, Belgium), based on a vertical nulling microbalance. Sample and reference crucibles (metal-coated quartz, 180 μL) were inserted in a humidity and temperature-controlled chamber.

Water was used as probe vapor. Samples were dried at 25 °C and 0% RH for 120 min and subsequently subjected to step changes from 0% RH up to 90% RH, and the opposite for desorption. The sample mass was allowed to reach equilibrium for a maximum time of 360 min at each step. The measurement also ended when the mass change was <0.02% for 15 min; only then did the RH increase. Sample weights were around 7.5 mg. The resulting data was exported into Microsoft^®^ Excel (Microsoft Corp., Redmond, WA, USA) for analysis. Sorption isotherms were determined at ten RH (%) values (0, 10, 20, 30, 40, 50, 60, 70, 80, and 90) and the isotherms were obtained at +5 °C and +25 °C.

#### 2.2.6. Powder X-ray Diffraction

The powders were analyzed by powder X-ray diffraction (PXRD) using an X-ray diffractometer (D8 Advance Eco Bruker^®^, Madison, WI, USA), equipped with a one-dimensional silicon detector (LynxEye, Bruker AXS, Billerica, MA, USA) and using Cu-Kα radiation (1.54 Å; 40 kV × 25 mA). Data were collected over the angular range of 4–40° 2θ, with a step size of 0.02° and a dwell time of 2 s. The percentage of amorphous form was calculated as 100% minus the crystalline phase content in the powder, as determined using the surface area ratio method.

#### 2.2.7. Particle Size Distribution

The volume particle size distribution (PSD) of the powders was determined by laser-diffraction, using a Malvern Mastersizer^®^ 3000 equipped with an Aero S dry powder disperser unit (Malvern Instruments Ltd., Worcestershire, UK).

Samples of dried powder (n = 3) were placed in the hopper of the disperser. During the measurement, 75% vibration was used and a pressure of 4 bar was achieved in order to individualize the particles. The dispersed material had a refractive index of 1.65 and an absorption index of 0.1. The refractive index of the dispersing medium (i.e., air) was set at 1.

#### 2.2.8. Bacteriophage Stock Preparation

Bacteriophage stocks were prepared using the double agar overlay method [17] with some modifications. The equivalency of Optical Density (OD) to the colony-forming unit (cfu)/mL was pre-determined for the *P. aeruginosa* strain PAO1 K. The bacteriophage/bacteria ratios were adjusted for efficient reproduction of each bacteriophage (*P. aeruginosa* LUZ19 and 14/1), in order to obtain a “web-pattern” on square Petri dishes (Greiner BioOne, International GmbH). The mix of equal volumes (250 μL) phage and bacteria in 15 mL tube was filled with molten agar (46 °C) up to 8 mL and overlaid on the surface of solid agar in the square petri dish. Liquid lysogeny broth (LB broth Miller, Animal Free Powder, VWR Chemicals, Leuven, Belgium) with 0.6% (soft layer) and 1.5% (solid layer) agar was used. After incubation at 32 °C for 18 h, the bacteriophages (soft layer) were harvested, centrifuged at 6000× *g* for 30 min, and further filtered using 0.22 μm membrane filters (Millex, Merck Millipore, Cork, Ireland). Then, the lysate was exposed to high-speed centrifugation at 35,000× *g* for an hour and a half, and the bacteriophage pellet was re-suspended in Dulbecco’s Phosphate-Buffered Saline (DPBS) without (*w*/*o*) Ca^2+^ and Mg^2+^ (Lonza, Verviers, Belgium) and stored at 4 °C. The titers for each propagated bacteriophage stock (2.0 × 10^11^ pfu/mL) were determined by the double agar overlay method [17].

#### 2.2.9. Bacteriophage Stability Titration

The contents of each test vial were reconstituted with DPBS *w*/*o* Ca^2+^ and Mg^2+^ to a final volume of 5 mL. Then, samples were homogenized by gentle mixing. The spot testing on the agar overlay technique [18], with some modifications, was used to determine the number of viable bacteriophages (pfu/mL) in the powder samples. Ten-fold serial dilutions of samples were made in 96-well microplates by adding 20 µL samples to 180 µL DPBS. All sample dilutions were carried out in triplicate. Bacterial lawns were made by mixing 300 µL of OD 0.3 host bacterial suspension with up to 8 mL of molten soft agar (0.7% LB, 46 °C) and overlaid onto pre-prepared square Petri dishes with 1.5% LB agar. A volume of 2 µL of diluted bacteriophage samples were spotted onto the soft agar surface, and test plates were air-dried in a biosafety cabinet and incubated upside down at 32 °C for 18 h. The number of pfu that were contained in 2 µL was expressed in pfu/mL. Every test plate included a standard bacteriophage sample with a pre-defined titer (pfu/mL) as a control of the titration process. Finally, the titer was converted from pfu/mL to pfu/mg, taking into account the initial mass of powder that was present in the vial [18].

#### 2.2.10. Stability Testing

Stability testing was performed according to the ICH harmonized guidelines, as described in the Q1A(R2) document [19]. Stability testing was conducted on the bacteriophage substances packaged in sealed vials for 12 months and tests were performed after 1 day, after 1, 3, 6, 9 months and 1 year at 4 ± 2 °C; 25 ± 2 °C and 60% RH; 30 ± 2 °C and 65% RH; and 40 ± 2 °C and 75% RH in climatic chambers (Weiss, Loughborough, UK).

Indeed, as described in ICH Topic Q 1 A (R2) “Stability Testing of new Drug Substances and Products”, the aim of the stability study is “to provide evidence on how the quality of a drug substance or drug product varies with time under the influence of a variety of environmental factors such as temperature, humidity, (…)”. Moreover, it’s also explained that the stability study should be conducted “on the drug substance packaged in a container closure system that is the same as or simulates the packaging proposed for storage and distribution.”

#### 2.2.11. Experimental Design

A response surface randomized I-optimal design, using Design Expert software (version 12.0.10.0; Stat-Ease Inc., Minneapolis, MN, USA), was devised to assess the effect of the spray-drying process parameters and formulations on powder process yield, residual moisture content (RMC), process outlet temperature (which is the temperature of the drying gas at the end of the column; T_out_), and loss of bacteriophage lytic activity (expressed as log_10_ pfu/mg).

The following parameters were selected for study: T_in_, spray gas flow, and L-isoleucine concentration. The settings for all the process parameters were based on preliminary studies, where T_in_ ranged from 60 °C to 80 °C (representing the lower and upper-temperature limits to achieve a low residual moisture content, and to ensure that thermal degradation of the formulation ingredients was minimized), spray gas flow ranged from 536 to 819 L/h, and L-isoleucine concentration from 10% to 40% *w*/*w* of the total solute content in the feed solution.

The experiments included 30 runs for both bacteriophages, targeted to evaluate and model the main effects, two- and three-way interactions, as well as quadratic and cubic terms. A block structure (with n = 5) was used to take into account the potential day effect, as a maximum of five runs could be performed on a single day. Within and between block randomization was applied.

For data accuracy, replicates were carried out at different steps: experimental runs, powder analysis (sampling) and titration.

## 3. Results and Discussion

Compared to freeze-drying, spray-drying is highly advantageous for the biopharmaceutical industry in terms of cost (e.g., lower footprint and continuous process) and product quality process efficiency (e.g., almost instantaneous, without freezing) [20,21,22]. Due to the inherent benefit of using spray-drying, this technique was selected as the preservation technique for our bacteriophage products. Regarding the formulation, lactose and trehalose are generally used to protect biological materials from desiccation in spray-drying. In their anhydrous amorphous form, both have high Tg (108 °C and 115 °C, respectively). Nonetheless, in peptide and protein drug formulations, the use of lactose is moot, due to its reducing properties [7]. In our case, the reducing functional groups of lactose may damage the integrity of the bacteriophages, leading to their inactivation. Therefore, trehalose was selected to protect the bacteriophages.

In addition to the use of sugars, other excipients are commonly used to improve the dispersibility of dry microparticles or to protect them from the deleterious effects of residual moisture by creating an outer shell around the particle. The amino acid most commonly used for this purpose is L-Leucine [23]. However, Mah et al. recently conducted a study to compare the effects of L-Leucine and L-isoleucine in reducing moisture-induced changes in spray-dried trehalose formulations [24]. They demonstrated the greater ability of L-isoleucine to overcome elevated humidity compared to L-Leucine consisting of samples of the same concentration. Therefore, L-isoleucine was selected for further usage.

In spray-drying, a dry powder was produced by atomization of a liquid dispersion through a nozzle into a hot drying gas flow. The characteristics of the solid particles (e.g., size distribution or residual moisture) can be modulated according to the parameters of the spray-dryer (e.g., feed rate, spray nozzle size, spray gas flow, drying gas temperature) [25]. These parameters are related and should be adapted depending on the context of the pharmaceutical environment and the aims of the development. Quality by Design (QbD) tools allow us to investigate both the formulation and the process design, understanding, and control simultaneously. One of the main tools used in QbD approaches is the Design of Experiment (DoE). In a DoE-based approach, selected input variables are varied at the same time in a structured way. Therefore, it is possible to assess their potential effects on output responses. Indeed, DoE allows for evaluation of the interactions between variables and their effect on various responses, as well as maximizing the information gained while minimizing the resources required [26].

In this study, an I-optimal design allowed us to determine the optimal conditions in which to spray-dry bacteriophages LUZ19 and 14-1, in a non-empirical way. The aim was to obtain LUZ19 powder and 14-1 powder with the following quality target product profile (QTPP): A minimal reduction of the bacteriophage titer (<1 log_10_ pfu/mg) after spray-drying, followed by a minimal residual moisture content for conservation and, finally, a maximal process yield.

### 3.1. Response Surface Analyzes

A randomized response surface study was implemented to investigate the effects of three parameters (T_in_, Spray gas flow, and L-isoleucine concentration) on the powder properties and preservation of bacteriophage lytic activity. This statistical method facilitated identification of the most significant factors influencing the residual activity of the bacteriophages after drying and the properties of the powders; that are, the critical quality attributes (CQAs).

**Figure 2:**
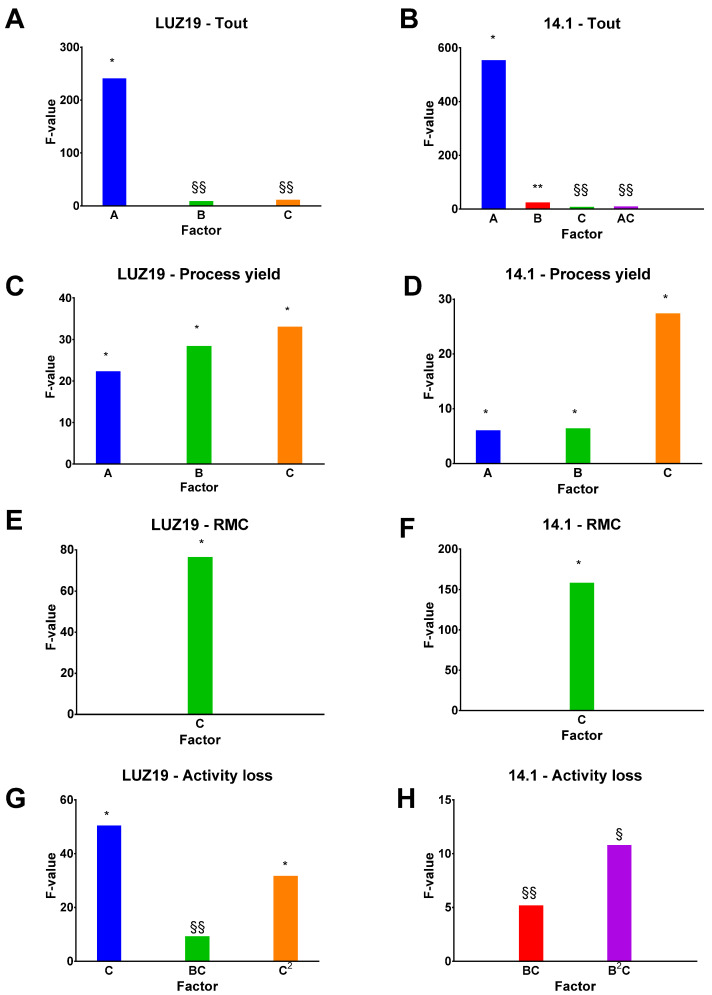
Bar plots showing F-values and associated *p*-values determining the statistical significance of the main effects and interactions for the response: (**A**) Outlet temperature (Tout) for the LUZ19 process; (**B**) Tout for the 14-1 process; (**C**) Process yield for the LUZ19 process; (**D**) Process yield for the 14-1 process; (**E**) Residual Moisture Content (RMC) for the LUZ19 process; (**F**) RMC for the 14-1 process; (**G**) Activity loss for the LUZ19 process; and (**H**) Activity loss for the 14-1 process. Only terms with *p*-values equal to or less than 0.05 were plotted. *, *p* < 0.0001; **, *p* < 0.001; §, *p* < 0.005; §§, *p* < 0.05. Factors: A: Main effect: Tin; B Main effect: Spray gas flow; C: Main effect: L-isoleucine concentration; BC: Two-factor interactions: Spray gas flow and L-isoleucine concentration; C2: Quadratic term modelling curvature of an ellipse in the response surface for L-isoleucine concentration; B2C: Cubic terms modelling inflected asymmetry for Spray gas flow and L-isoleucine concentration.

Regression analysis of the data was carried out and models for each output parameter were obtained. An analysis of variance (ANOVA) was carried out, in order to evaluate the significance of the terms of the model. Each model had an F-value much greater than 1, which means that the models were significant, and *p*-values lower than 0.05, which indicated that the model terms were significant (Figure 2). Indeed, the F-Value is the ratio of the Mean Square Total divided by Mean Square Residual and, so, it indirectly indicates that the model term effect is very sharp, compared to the residual error effect. On the other hand, *p* < 0.05 indicates that there is less than 5% probability of falsely detecting a significant effect or, correspondingly, there is more than 95% confidence that the selected model term will have a significant effect on the corresponding response. Moreover, the R^2^ values were lower than 0.2 and, thus, in reasonable agreement with the adjusted R^2^ values (Table S1) [27]. Furthermore, the Adeq precision measures the signal-to-noise ratio. Ratios greater than 4 were obtained, which means that the signals were adequate. The models were statistically significant relative to noise and, hence, could be used for further exploration and prediction.

T_out_ ranged between 29 °C and 40 °C and, as expected, was impacted by T_in_ (Figures S1 and S2). Moreover, a higher spray gas flow decreased T_out_, while a higher L-isoleucine ratio increased it. Indeed, a higher spray gas flow decreased T_out_, due to the additional amount of cold gas that needed to be heated up [28]. Higher L-isoleucine ratios were found to increase T_out_, probably due to the increase in less hydrophilic amino acid concentration, which induced the formation of a more hydrophobic layer due to the presence of greater amounts of L-isoleucine at the surface of the particles. Such hydrophobic interactions facilitate the evaporation of water at the surface of the particles. Lower energy is used for the drying, which leads to a slight increase of the Tout [29,30,31].

The process yield output ranged from 72% to 85% *w*/*w*, and the most influential input parameter was the L-isoleucine concentration (Figures S3 and S4). The higher the L-isoleucine concentration, the lower the process yield, within the explored range. This observation was in contrast with previous reports in the scientific literature [32]. However, it could be explained by the electrostatic behavior of powder. Indeed, the percentage of L-isoleucine determines the level of powder dryness (Figures S5 and S6) and, therefore the generation of electrostatic charges. Indeed, the drier the powder, the higher the charge of the powders [33,34,35]. As a consequence, the dry particles could be held up to the glassware walls of the spray-dryer; and, more particularly, the glassware walls of the cyclone, where the powder separates from the drying air. We also observed a drop in the quantity of powder collected in the vessel collector. The spray gas flow had a negative impact on the process yield (%) as well, in contradiction with the literature [31]. An increase in the nozzle gas flow caused an increase in the atomization energy, leading to the production of narrowed droplets. These droplets dry into smaller particles, which are more easily captured by the centrifugal force in the cyclone [36]; however, this depends on the size limit of the droplets/particles generated. Below a certain size (very small particles), the centrifugal forces generated at the level of the cyclone are no longer sufficient to allow the separation of the particles from the air stream, and the particles will be returned to the level of the filter at the outlet of the equipment [37]. It was also assumed that, once the droplets had escaped the near nozzle region, they were exposed to the aerodynamic forces imposed by the drying gas flow. Therefore, their trajectories were altered or they impacted a sidewall of the drying column, causing a potential loss of product yield [38]. In contrast, T_in_ had a positive impact on the process yield (%). Indeed, T_in_ is correlated with the temperature at the end of the column (T_out_) (Figures S1 and S2) [31]. As a high T_out_ promotes the formation of powder with low residual moisture, the process yield increased (but not too low, as the yield would decrease with the generation of electrostatic charges or if the Tg of the product was exceeded at the bottom of the column, then a loss could occur, as the product would stick to the wall of the cyclone) [39].

RMC is an important parameter that influences the physical stability of amorphous spray-dried powders. The level of moisture could affect particle size distribution of the dried sample, as well as the potential crystallization of the excipients during long-term storage. Such crystallization, which is known to occur due to plasticization of the amorphous phase, could inactivate the bacteriophages [15]. The RMC ranged from 2.1 to 4.9% *w*/*w* and was significantly affected by the L-isoleucine concentration (Figures S5 and S6). Higher L-isoleucine concentrations reduced the RMC in powders. Indeed, the increase in less hydrophilic amino acid concentration induced the formation of a more hydrophobic layer, due to the presence of greater amounts of L-isoleucine at the surface of the particles, thus promoting the evaporation of water. On the other hand, L-isoleucine is important for powder conservation. It has been suggested that L-isoleucine, similarly to L-Leucine, preferentially enriches the surface of the particles during the drying process [36,37,38,39] and, due to its hydrophobic nature, can act as a barrier to moisture and slow down the progress of moisture into the particles [24].

**Figure 3:**
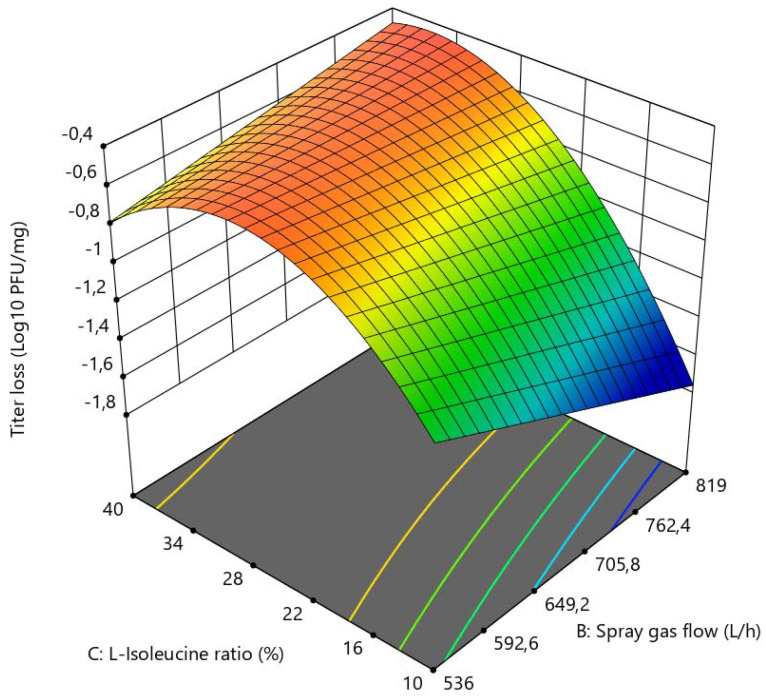
3D surface graph showing bacteriophage LUZ19 activity (log_10_ pfu/mg), in relation to L-isoleucine ratio (%) and spray gas flow (L/h).

**Figure 4:**
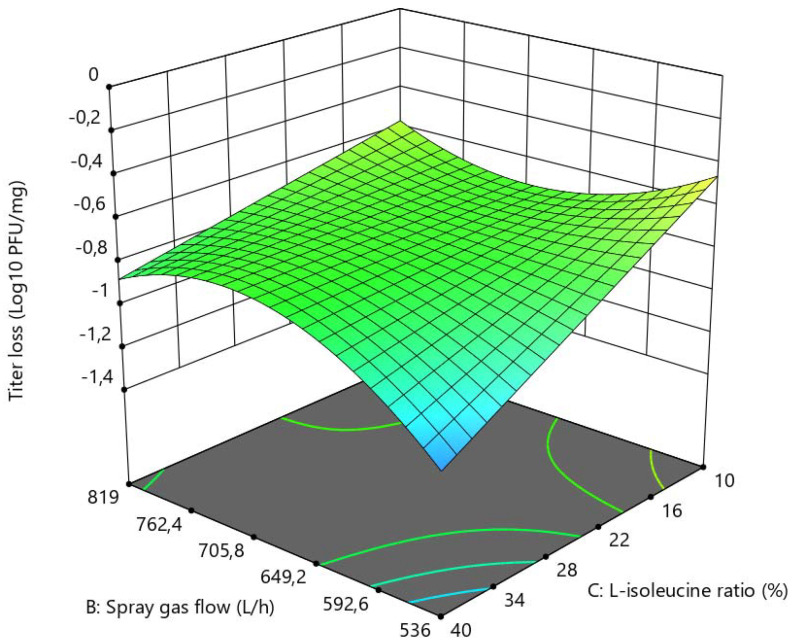
3D surface graph showing bacteriophage 14-1 activity (log_10_ pfu/mg), in relation to L-isoleucine ratio (%) and spray gas flow (L/h).

L-isoleucine and spray gas flow had a significant impact on the titer reduction (log_10_ pfu/mg) of LUZ19 after spray-drying (Figure 3). The higher the concentration of L-isoleucine, the better the bacteriophage titer was maintained after drying (with a quadratic effect). However, high concentrations of L-isoleucine were no longer supportive when the spray gas flow was low. The combination of low concentrations of L-isoleucine and high spray gas flow led to decreasing bacteriophage titers after drying. In contrast to the bacteriophage LUZ19, both spray gas flow and L-isoleucine concentration had only slight effects on bacteriophage 14-1 (Figure 4). Interestingly, L-isoleucine concentration had a more negative effect on bacteriophage 14-1 than on bacteriophage LUZ19. Furthermore, as explained previously, the RMC within the powder was inversely proportional to the concentration of L-isoleucine, according to which we hypothesized that certain bacteriophages would need a certain residual humidity within the powder after drying according to their morphology. Indeed, it can be suggested that, in a powder of amorphous structure, the myoviridae phages (which are characterized, among other things, by a larger size than the podoviridae phages) need more residual moisture within the powder to be stabilized without being inactivated. It would be interesting, subsequently, to verify this hypothesis with other podoviridae and myoviridae bacteriophages.

For both bacteriophages, the spray gas flow was beneficial, in contrast to the conclusion of Vandenheuvel et al. [7], who hypothesized that an elevated atomizing airflow resulted in an increased reduction of bacteriophage titer. Moreover, in their study, they suggested that bacteriophages with delicate structures—for instance, those with long and rigid tails and tail fibers—are more sensitive to shear forces. In the present study, however, bacteriophage 14-1 (a myovirus), was not more inactivated than bacteriophage LUZ19 (a podovirus). Consequently, our results suggest that the influence of the drying process is specific to individual bacteriophages, rather than bacteriophage particle morphology.

### 3.2. Determination of Optimal Spray-Drying Conditions

**Figure 5:**
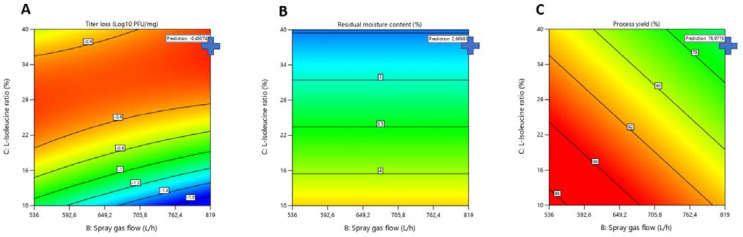
LUZ19 contour plot: Representation of Titer loss (**A**); Residual Moisture Content (**B**); and Process yield (**C**) against combinations of Spray gas flow and L-isoleucine ratio factors.

**Figure 6:**
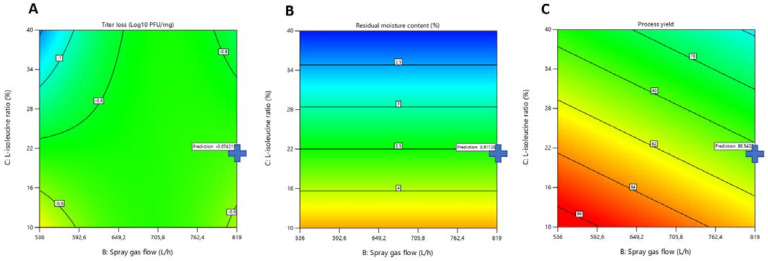
14-1 contour plot: Representation of Titer loss (**A**); Residual Moisture Content (**B**); and Process yield (**C**) against combinations of Spray gas flow and L-isoleucine ratio factors.

Using a DoE approach, a model for each bacteriophage was developed. From these models, input parameters were selected, in order to be able to produce a powder with interesting characteristics, in terms of output properties (QTTP). More particularly, we decided to increase the yield of the process (%) as much as possible, to decrease the residual moisture content (% *w*/*w*; in view of long-term storage stability), and to limit the titer reduction (log_10_ pfu/mg). The most important output was bacteriophage titer reduction (log_10_ pfu/mg). Therefore, we decided to focus on a final dried product characterized by a minimal reduction of the bacteriophage titer (<1 log_10_ pfu/mg), followed by minimal residual moisture content and, finally, a maximal process yield. For the two bacteriophages, the optimal parameters of the drying process—the inlet temperature and the spray-gas flow—were the same (80 °C and 819 L/h, respectively). However, for the L-isoleucine concentration, the optimum concentration for drying LUZ19 was 36.7% *w*/*w*, while that for 14-1 was 20.6% *w*/*w* (Figure 5 and Figure 6).

**Figure 7:**
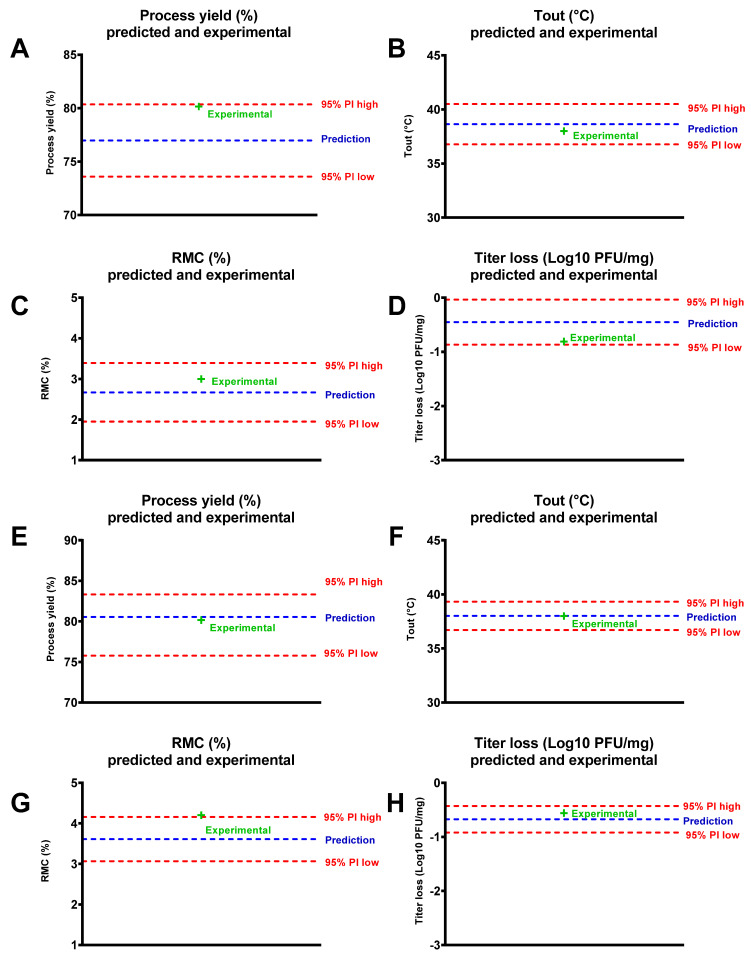
Experimental optimal response compared to prediction, 95% prediction interval (PI) low and 95% PI high for LUZ19 (**A**–**D**) and 14-1 (**E**–**H**), for each response.

Then, the optimized powders were produced and the experimental results were compared with the model predictions for both the 95% prediction interval (PI) low and 95% PI high (Figure 7). The results showed that, for each output, the results fit the predictions well (e.g., for LUZ19 and 14-1 optimized powders, 38 °C for T_out_ was predicted, which was the exact value of the real T_out_; for RMC, 2.85% was predicted for LUZ19 and 3.6% for 14-1, the real values were 3% and 4.2%, respectively).

### 3.3. Characterization of Optimal Powders

#### 3.3.1. SEM

**Figure 8:**
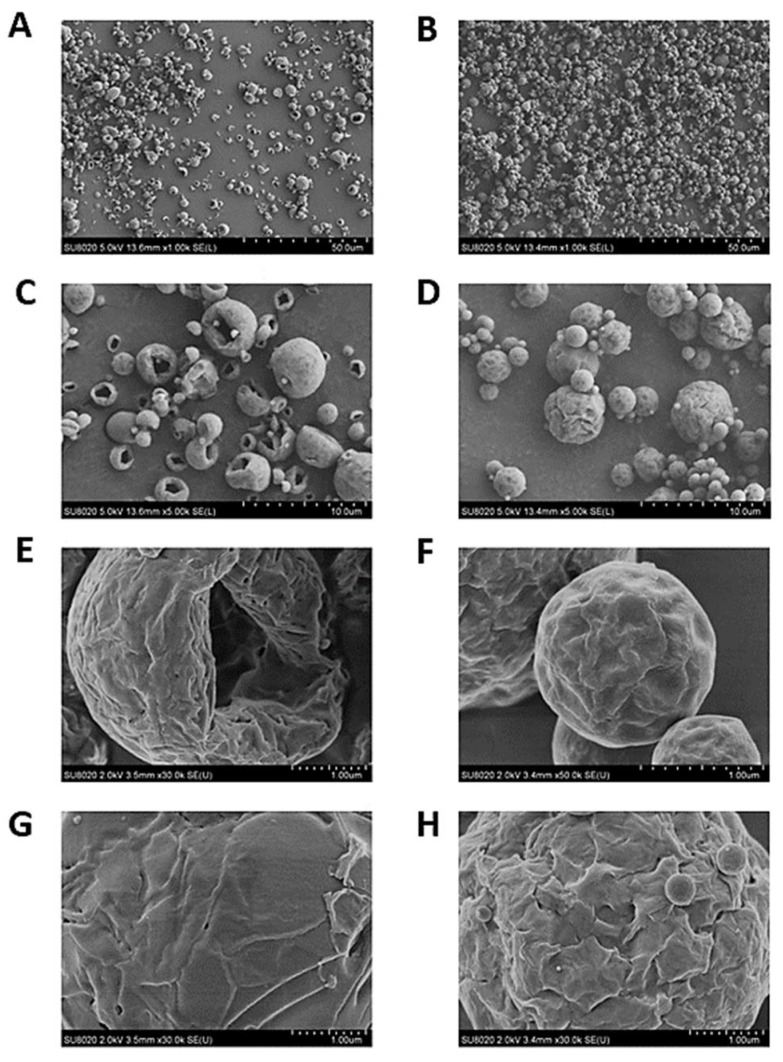
Representative scanning electronic micrographs of LUZ19 (**A**,**C**,**E**,**G**) and 14-1 (**B**,**D**,**F**,**H**) powders.

The pictures obtained by SEM show at the smallest magnification (×1000), homogeneous particles for the optimized powder particles containing LUZ19 and 14-1 (Figure 8A and 8B, respectively). It was observed at a magnification five times higher than the particles of the powder containing LUZ19 (Figure 8C) are folded particles contrary to the particles containing 14-1, which are spherical (Figure 8D). For LUZ19, the dried particles had a lower spherical appearance than those of 14-1 This difference is even more visible when increasing the magnification six more times (Figure 8E,F).

This could be explained by the Peclet number (*Pe*) [29]. Indeed, the optimized LUZ19 powder contained more L-Isoleucine than the optimized 14-1 powder (36.7% *w*/*w* and 20.6% *w*/*w*, respectively).

The Peclet number (*Pe*) is defined by Equation (1) [29], where *D* represents the diffusion rate of the dissolved solute and *K* is the surface evaporation rate:

(1) $$Pe=\frac{K}{8D}.$$

The evaporation rates of the spray-dried formulations containing D-(+)-Trehalose dihydrate and L-isoleucine were similar, as they were exposed to the same drying conditions. However, D-(+)-Trehalose dihydrate and L-isoleucine have different molecular weights (378.33 and 131.17 g/mol, respectively). Thus, it can be assumed that the diffusion rate of L-isoleucine is greater than that of D-(+)-Trehalose dihydrate. Therefore, the Peclet numbers for D-(+)-Trehalose dihydrate and L-isoleucine would also be very different. Due to its high Pe, during drying, L-isoleucine precipitated on the surface of the droplets, forming a hydrophobic layer, which interfered with the diffusion of water and induced the formation of corrugated particles. This sometimes led to the formation of hollow grains [40] (Figure 8E).

We can also observe that the particles are corrugated but particles containgLUZ19 showed a less rough surface than particles containing 14-1 (Figure 8G,H).

#### 3.3.2. Particle Size Distribution

Particle size and shape may affect many important physical properties, manufacturing processability, and quality attributes of dry powder. A Dx(50) of 2 micrometers for LUZ19 and 14-1 spray-dried particles was observed (Table 1). Small and hydrophobic particles usually allow for enhanced liberation, dispersion, and bioavailability of the active pharmaceutical ingredients.

#### 3.3.3. Dynamic Vapor Sorption

DVS was used to evaluate the effect of moisture on the solid-state stability of the powders. For this experiment, only LUZ19 and 14-1 powders produced using optimal spray-drying conditions were evaluated at +5 °C (and, additionally, at 25 °C for LUZ19, in order to compare both storage temperatures; see Figure S7). Optimal LUZ19 and 14-1 powders showed similar DVS profiles: both were characterized by the presence of a loop and their sorption isotherms presented an inflection point at ~40% RH, reaching about 10% mass increment of their initial weight at 90% RH. At ~60% RH, the DVS curves were characterized by mass changes varying from ~10% to 5% *w*/*w* of their initial weight. The critical relative humidity was found to be around 40% RH (inflection point), representing the maximum relative humidity that the sample can withstand. Then, a decrease in the mass of the sample was observed. The critical relative humidity was exceeded, resulting in recrystallization of the sample and the expulsion of absorbed water. Subsequently, when the sample was subjected to a reduction in relative humidity (desorption isotherm), the mass of the sample did not change, as it was in crystalline form. Above 60% RH, adsorption became irreversible due to crystallization, and the sample permanently kept its water mass. At +25 °C, there was no significant difference with regard to moisture recovery.

### 3.4. Stability Study

**Figure 9:**
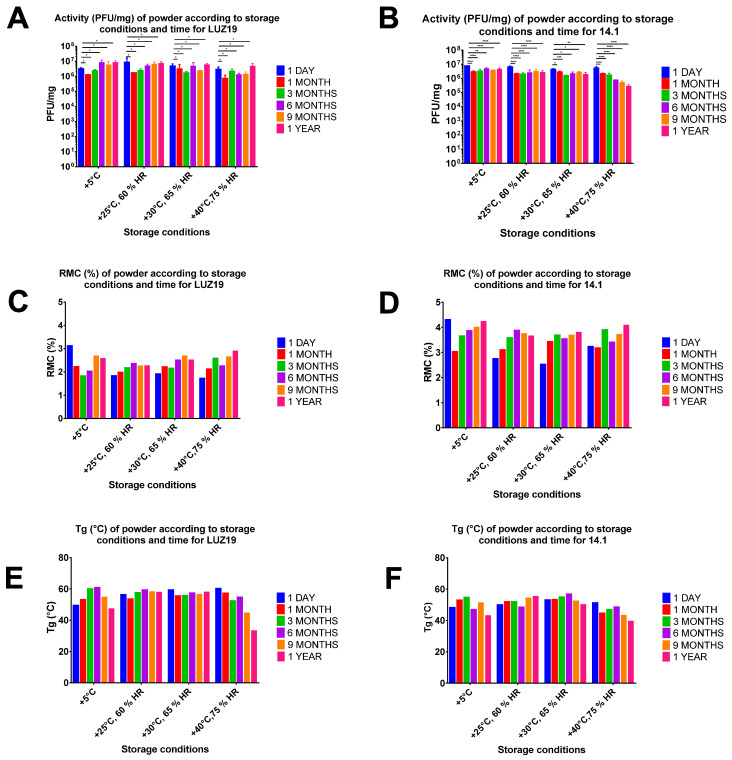
Evolution of bacteriophage activity (pfu/mg) in the optimized spray-dried powders containing LUZ19 (**A**) and 14-1 (**B**), as well as RMC (%) and Tg (°C) evolution over time for optimized spray-dried powders containing LUZ19 (**C**,**E**) and 14-1 (**D**,**F**) under different storage conditions for up to one year. A Dunnett’s multiple comparisons test was used for the statistical analysis. °, *p* > 0.05; *, *p* < 0.05; **, *p* < 0.005; ***, *p* < 0.0005; ****, *p* = 0.0001.

For the LUZ19 bacteriophage, no significative difference in activity was observed over time under each ICH condition and between each factor (Figure 9A), but with an increase of activity after 6 months at a storage temperature of +5 °C. An increase in activity was also observed between months 3 and 6, at +5 °C, +25 °C, and +30 °C. We hypothesized that these increases of activity during storage were due to the possibility that a fraction of the bacteriophage particles aggregated during the drying process, and that a certain period of storage worked in favor of dispersing these aggregates more effectively during the reconstitution. The phenomenon of bacteriophage aggregation has been reported to be associated with bacteriophage survival, having a moderate inhibitory effect on bacteriophage infectivity [41]. Mattle et al. have shown that MS2 bacteriophage aggregates were more resistant to micro-environmental changes than their dispersed analogues [42]. Aggregation could even be considered as a means for optimizing the reproduction of lytic bacteriophages [43]. In our study, storage time might have worked as a disaggregation factor, allowing the bacteriophage particles to disperse and, correspondingly, for the titer to recover.

For 14-1 (Figure 9B), significant differences were observed between T0 and one year of storage; however, the difference was lower than 1 log10 and the curve indicated that there was stabilization, except at +40 °C and 75% RH. It appears that the decrease in activity was not caused by crystallization, but by sensitivity to temperature, as the percentage of crystalline form did not differ over time. Indeed, XRD data (Figures S8 and S9) showed that all the phage powders remained partially crystalline with diffraction peaks from crystalline L-isoleucine at 6.3°, 12.7°, 18.5°, 19.1°, 25.1°, 25.5°, 32.0° and 32.4° [24]. The presence of phages does not affect the XRD pattern because the phage content in the spray dried powder is extremely low compared to excipients [15].

Moreover, XRD diagrams (Figures S8 and S9) showed a higher percentage of crystalline form for LUZ19 than for 14-1 powder (around 50% and 30% respectively). This could be explained by the differences in formulation. Indeed, the L-isoleucine ratio was more important in the LUZ19 optimized formulation than the 14-1 formulation (36.7% *w*/*w* and 20.6% *w*/*w*, respectively), while our experiment showed a negative influence of L-isoleucine on RMC (Figures S5 and S6). Furthermore, a lower value of Tg can be correlated to higher moisture content, as inferred from the Gordon–Taylor equation [44].

The difference in the maintenance of LUZ19 and 14-1 bacteriophage activities at 40 °C could be explained by two hypotheses. From one side, when the difference between the storage temperature and the Tg of the formulation exceeds 20 °C, the formulation is stable over time (regardless of the concentration of L-Isoleucine). From the other side, at +40 °C the activity is stable when the concentration of L-isoleucine is 36.7% but not at 20.6%. L-Isoleucine seems to have a protective role regarding the phage activity. It would be interesting to investigate the activity maintenance over longer time periods and to conduct extensive comparative stability study without and with different concentration of L-isoleucine to better understand its protective role but also with other phages to see if it is phage-specific. Moreover, establishing a scientific base for longer shelf times of dried bacteriophage products at 40 °C will allow for their distribution to developing countries, where the available refrigeration capacity is limited [45].

## 4. Conclusions

Optimized biologically active and stable dry powders containing *Podoviridae* or *Myoviridae P. aeruginosa* bacteriophages were produced by spray-drying. A Design of Experiment approach was used, including a response surface, in order to understand the relationships between process/formulation parameters and CQAs. An optimal set of parameters was determined using the obtained models, which was subsequently used in preservative spray-drying runs. For the bacteriophage LUZ19, the retention of activity after drying was proportional to the concentration of L-Isoleucine (with a quadratic effect). However, activity decreased at high L-Isoleucine concentrations when the spray gas flow was low. In contrast, at low L-Isoleucine concentrations, bacteriophage activity after drying decreased with a greater spray gas flow. Unlike LUZ19, for the bacteriophage 14-1, the effect of the spray gas flow, as well as the L-Isoleucine concentration, was much less pronounced. However, we observed a negative effect of L-isoleucine concentration and a positive effect of the spray gas flow on the activity of the bacteriophage 14-1. Finally, the powders remained stable for a minimum 1 year under different ICH conditions. To our knowledge, this is the first study in which bacteriophages have been spray-dried with isoleucine, with excellent biological stability up to 40 °C and 75% RH.

## Figures and Tables

**Table 1:** Particle size analysis of optimal LUZ19 and 14-1 spray-dried powders.

	Dx (10) (µm)	Dx (50) (µm)	Dx (90) (µm)	D[4,3] (µm)	Span
			LUZ19		
Average	0.912	2.180	5.100	1.677	1.720
Standard deviation	0.015	0.056	0.529	-	-
			14-1		
Average	0.476	2.079	4.857	2.843	2.062
Standard deviation	0.025	0.056	0.476	-	-

## Data Availability

All data generated and analyzed during this study are included in this article and its supplementary information files.

## Supplementary Materials

The following are available online at www.mdpi.com/article/10.3390/v13101926/s1, Figure S1. Temperature outlet, one-factor effect graphs with 95% CI bands for the LUZ19 experiments.; Figure S2. Temperature outlet, 3D surface graph for 14-1.; Table S1. Fit statistics for the models.; Figure S3. Process yield, one-factor effect graphs with 95% CI bands for the LUZ19 experiments.; Figure S4. Process yield, one-factor effect graphs with 95% CI bands for the 14-1 experiments.; Figure S5. RMC, one-factor effect graphs with 95% CI bands for the LUZ19 experiments.; Figure S6. RMC, one-factor effect graphs with 95% CI bands for the 14-1 experiments.; Figure S7. Dynamic Vapor Sorption (DVS) analyses for optimized spray-dried powder containing LUZ19 (A & B) and 14-1 (C&D) at +5 °C, as well as LUZ19 at +25 °C (E & F).; Figure S8. XRD profiles of optimized spray-dried powder containing LUZ19 stored at 5 °C (A) and 40 °C, 75% RH (B) over time, and at day 1 (C) and at 1 year (D) storage under different ICH conditions.; Figure S9. XRD profiles of optimized spray-dried powder containing 14-1 stored at 5 °C (A) and 40 °C, 75% RH (B) over time, and at day 1 (C) and at 1 year (D) storage under different ICH conditions.
