# Supplementary material for: A Design of Experiment Approach to Optimize Spray-Dried Powders Containing Pseudomonas aeruginosa Podoviridae and Myoviridae Bacteriophages
Source: Viruses. 2021 Sep 24;13(10):1926. doi: 10.3390/v13101926 (PMC8541621; doi:10.3390/v13101926)
Supplement: Supplementary file 1 [file viruses-13-01926-s001.zip › viruses-1396471-supplementary.pdf]

viruses

Supplementary Material

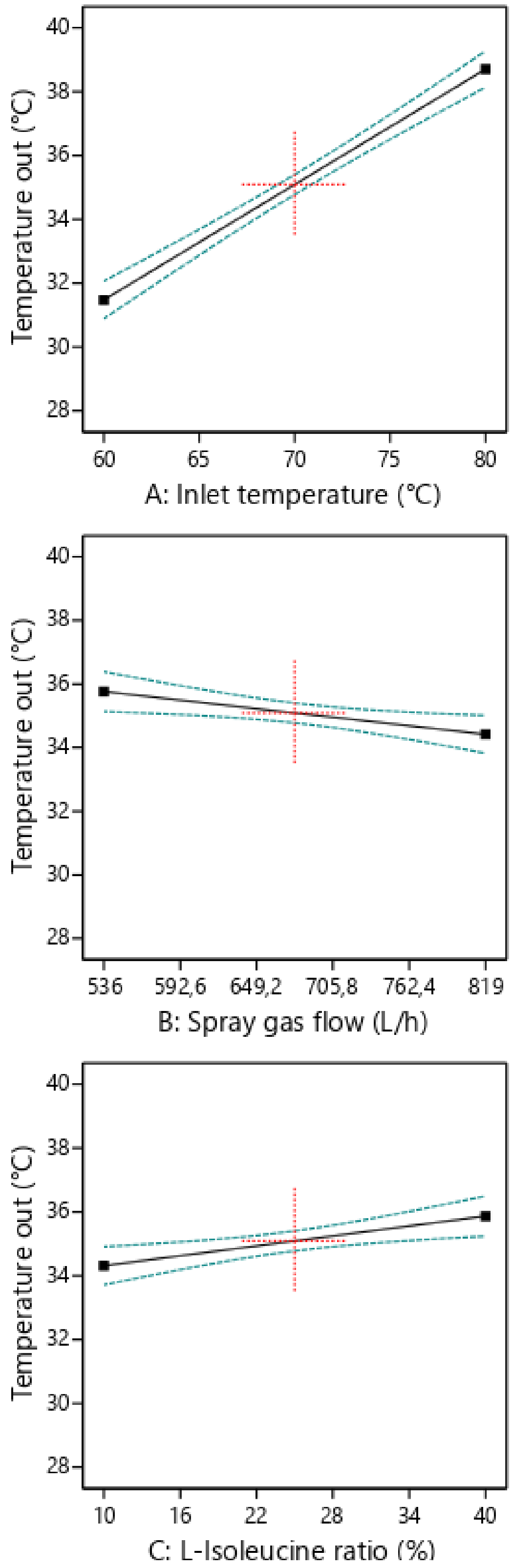

Figure S1. Temperature outlet, one-factor effect graphs with 95% CI bands for the LUZ19 experiments.

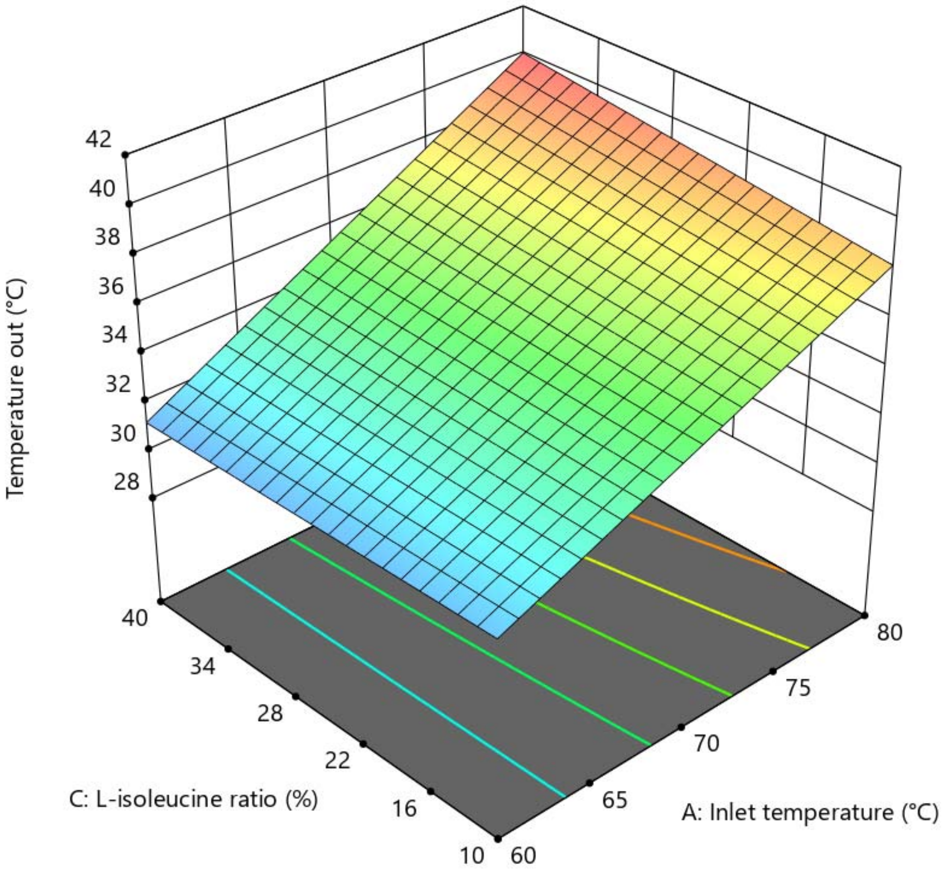

Figure S2. Temperature outlet, 3D surface graph for 14-1.

Table S1. Fit statistics for the models.

	**LUZ19**	**14-1**
Outlet temperature (°C)		
R^2	0.9248	0.9676
Adjusted R^2	0.9141	0.9608
Predicted R^2	0.8448	0.9213
Adeq precision	24.3143	33.333
Process yield (%)		
R^2	0.7924	0.7242
Adjusted R^2	0.7628	0.6782
Predicted R^2	0.5875	0.4153
Residual moisture content (%)		
R^2	0.7835	0.8870
Adjusted R^2	0.7732	0.8814
Predicted R^2	0.6215	0.7810
Loss of activity (Log10 PFU/mg)		
R^2	*0,7930*	*0,6612*
Adjusted R^2	*0,7516*	*0,5721*
Predicted R^2	*0,5720*	*0,2424*

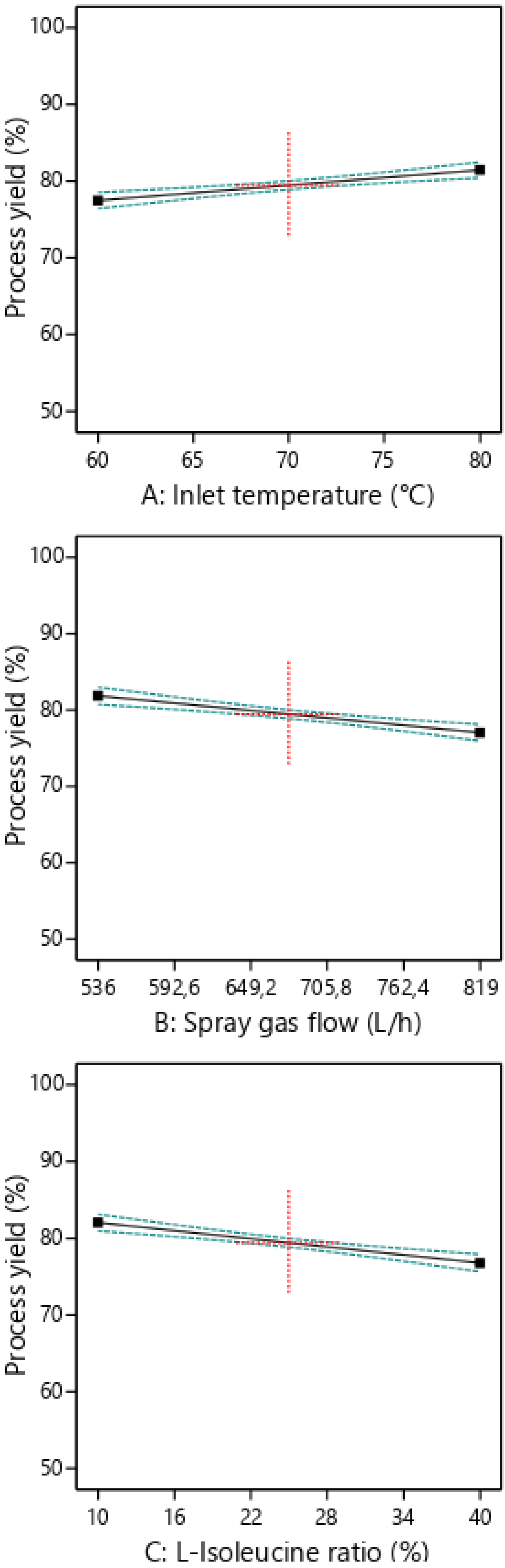

Figure S3. Process yield, one-factor effect graphs with 95% CI bands for the LUZ19 experiments.

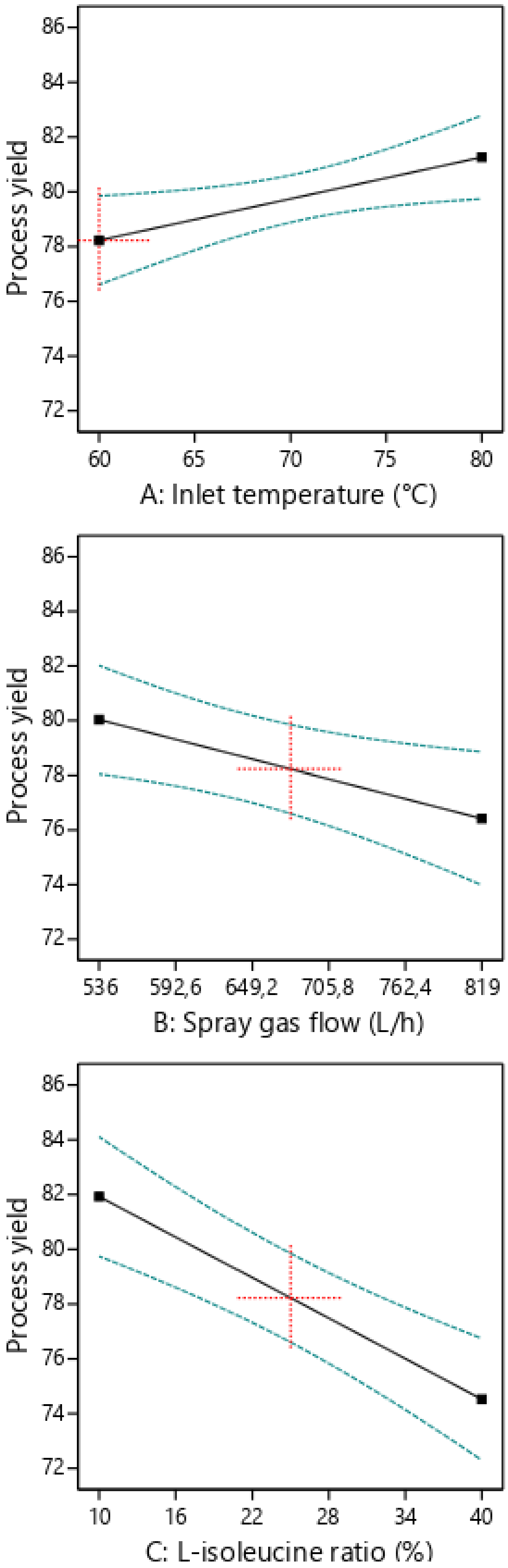

Figure S4. Process yield, one-factor effect graphs with 95% CI bands for the 14-1 experiments.

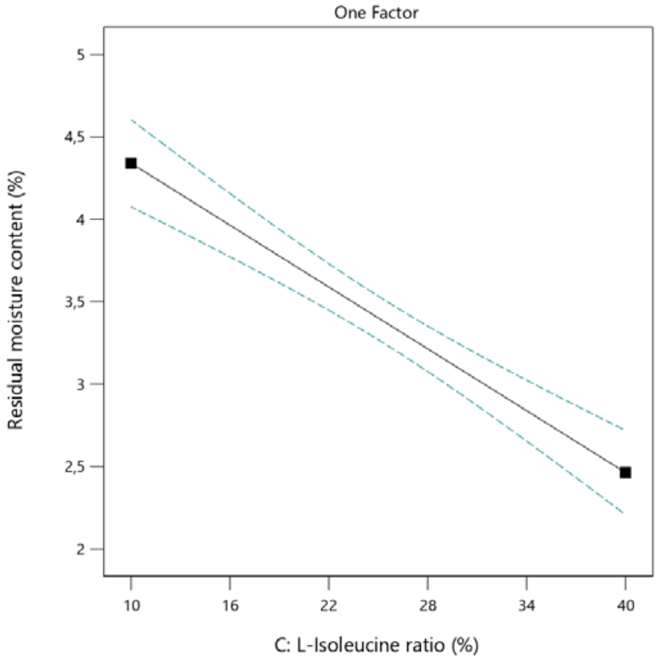

Figure S5. RMC, one-factor effect graphs with 95% CI bands for the LUZ19 experiments.

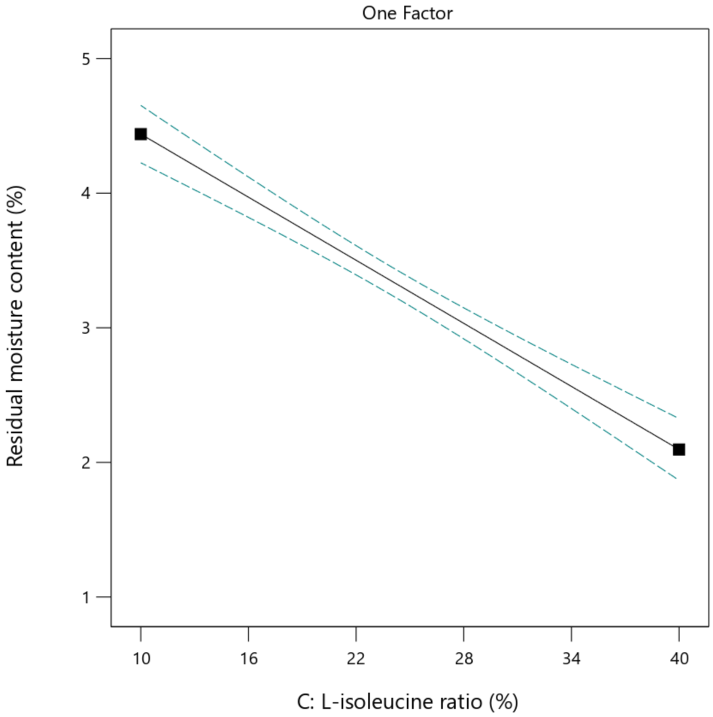

Figure S6. RMC, one-factor effect graphs with 95% CI bands for the 14-1 experiments.

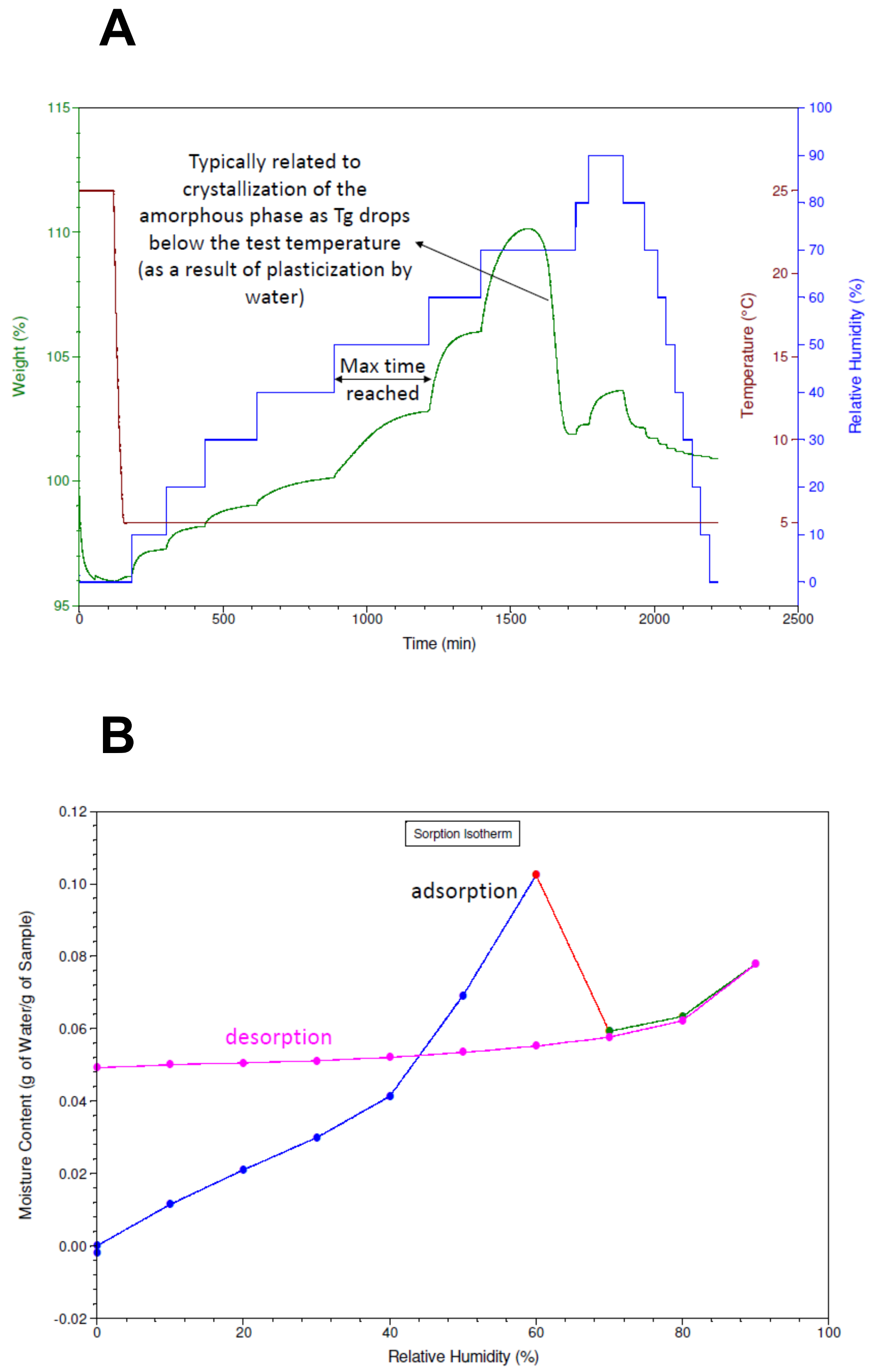
A
Typically related to crystallization of the amorphous phase as Tg drops below the test temperature (as a result of plasticization by water)
Max time reached
Weight (%)
Temperature (°C)
Relative Humidity (%)
Time (min)
B
Sorption Isotherm
adsorption
desorption
Moisture Content (g of Water/g of Sample)
Relative Humidity (%)

C

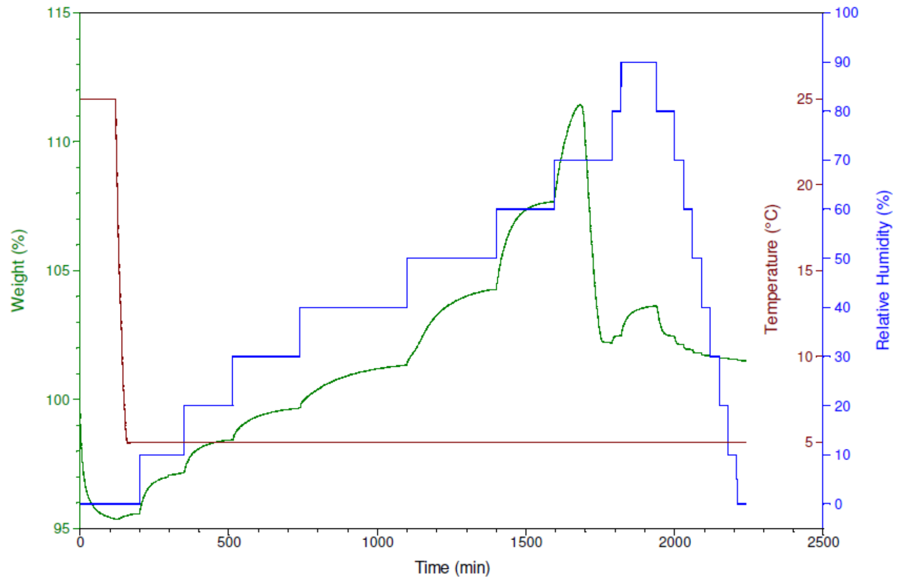

Weight (%)
Temperature (°C)
Relative Humidity (%)
Time (min)

D

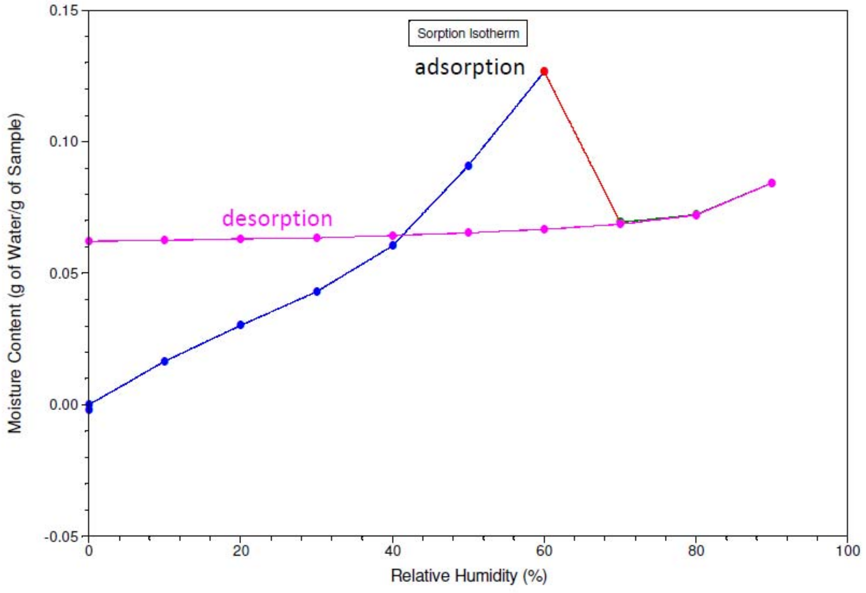

Sorption Isotherm
adsorption
desorption
Moisture Content (g of Water/g of Sample)
Relative Humidity (%)

E

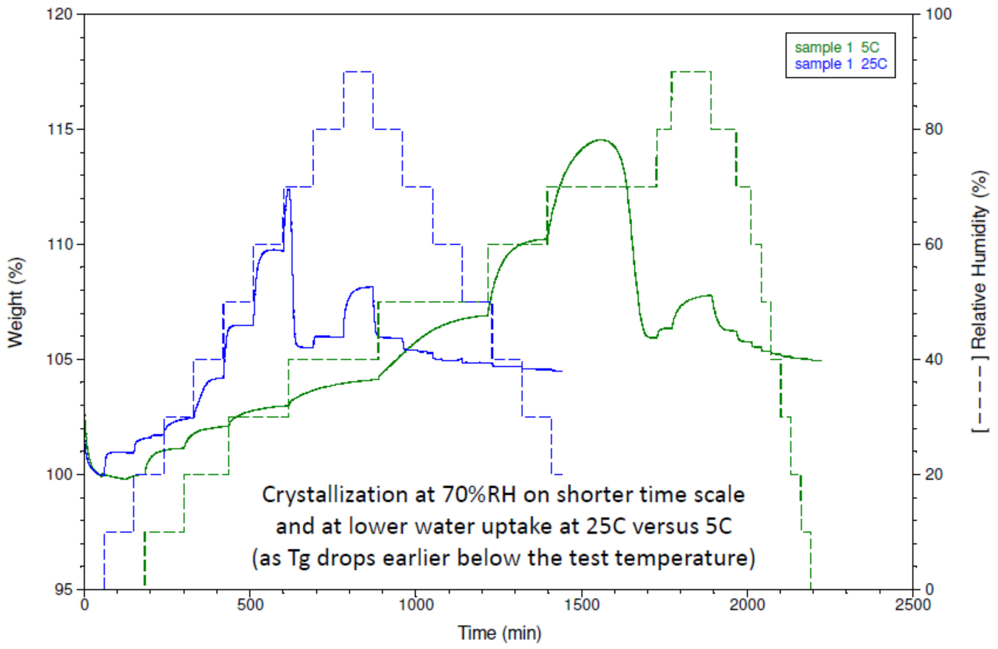

F

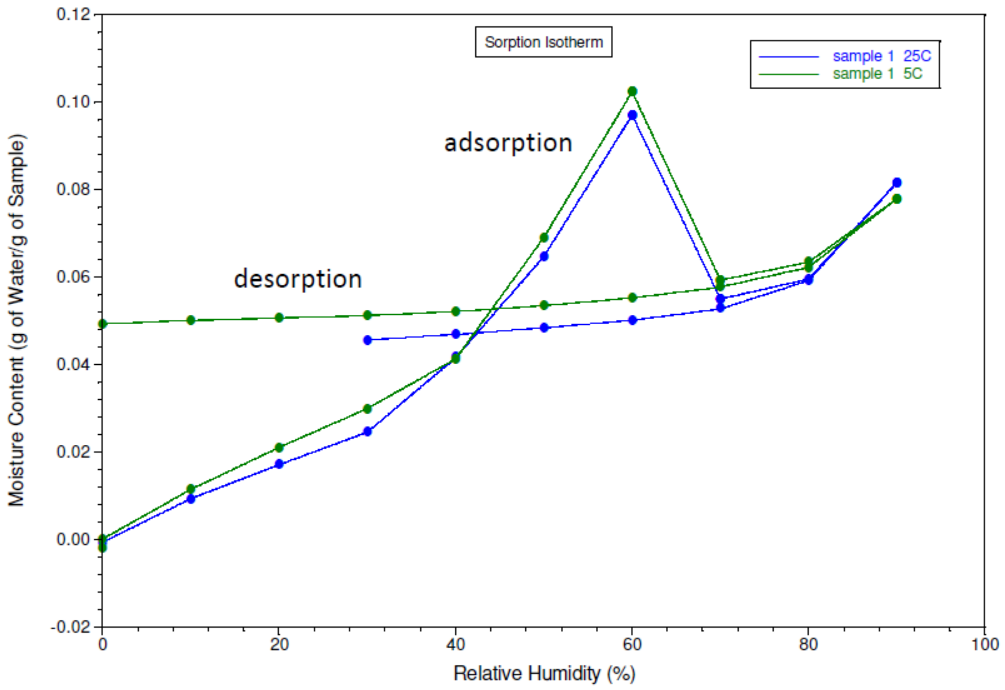

Figure S1. Dynamic Vapor Sorption (DVS) analyses for optimized spray-dried powder containing LUZ19 (A & B) and 14-1 (C&D) at +5 °C, as well as LUZ19 at +25 °C (E & F).

A

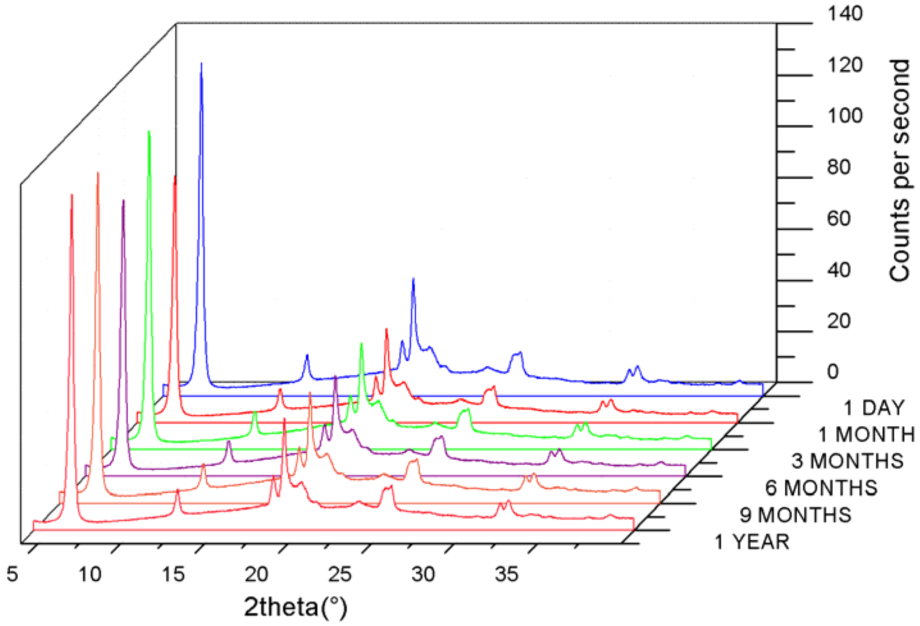
140
120
100
80
60
40
20
0
Counts per second
1 DAY
1 MONTH
3 MONTHS
6 MONTHS
9 MONTHS
1 YEAR
5
10
15
20
25
30
35
2theta(°)

B

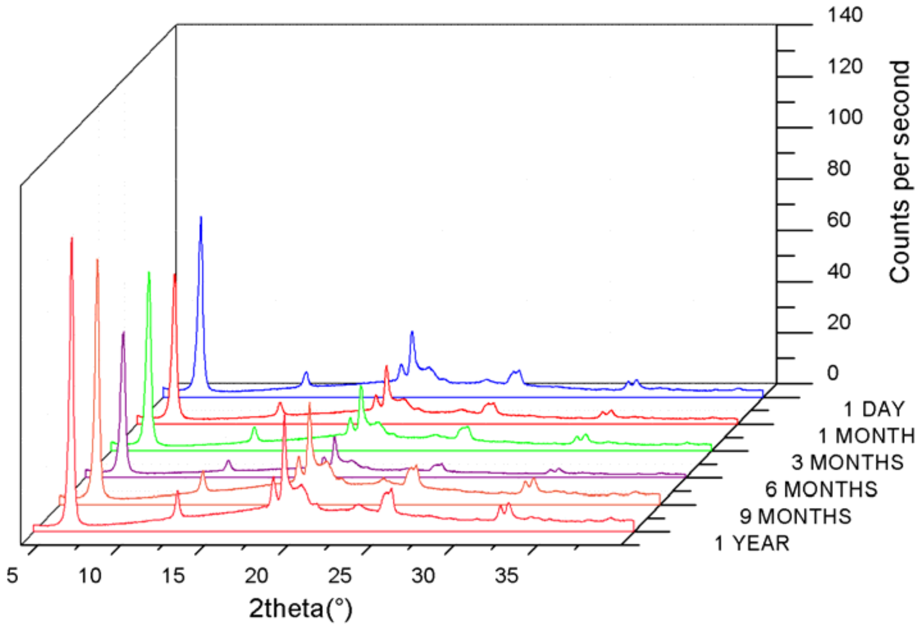
140
120
100
80
60
40
20
0
Counts per second
1 DAY
1 MONTH
3 MONTHS
6 MONTHS
9 MONTHS
1 YEAR
5
10
15
20
25
30
35
2theta(°)

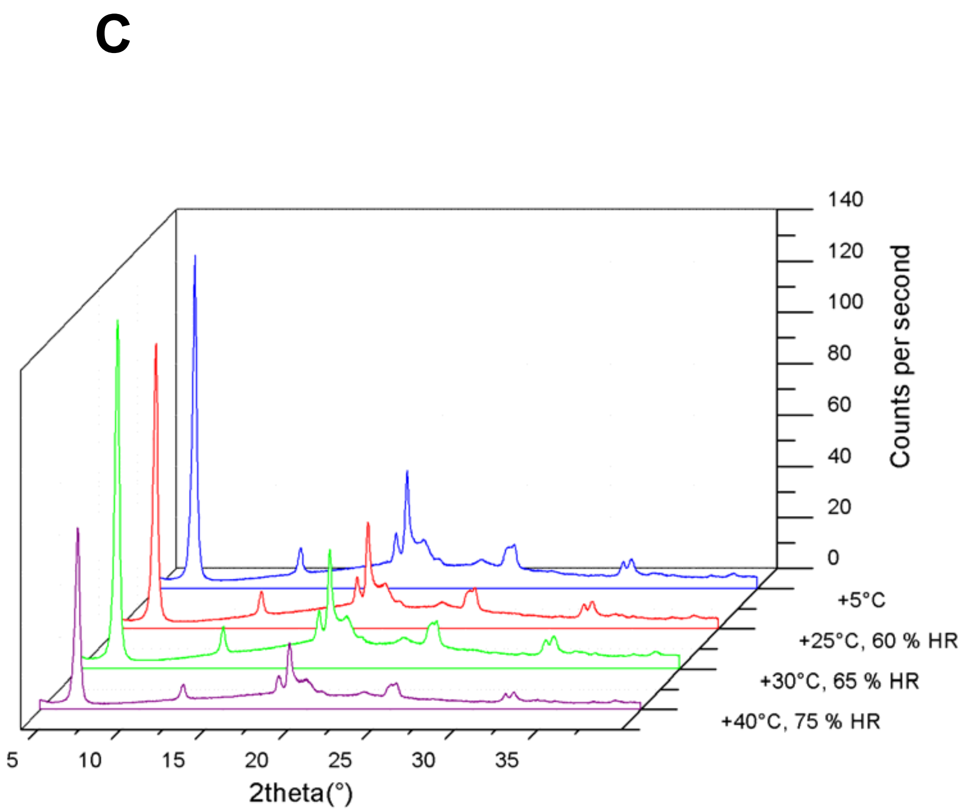

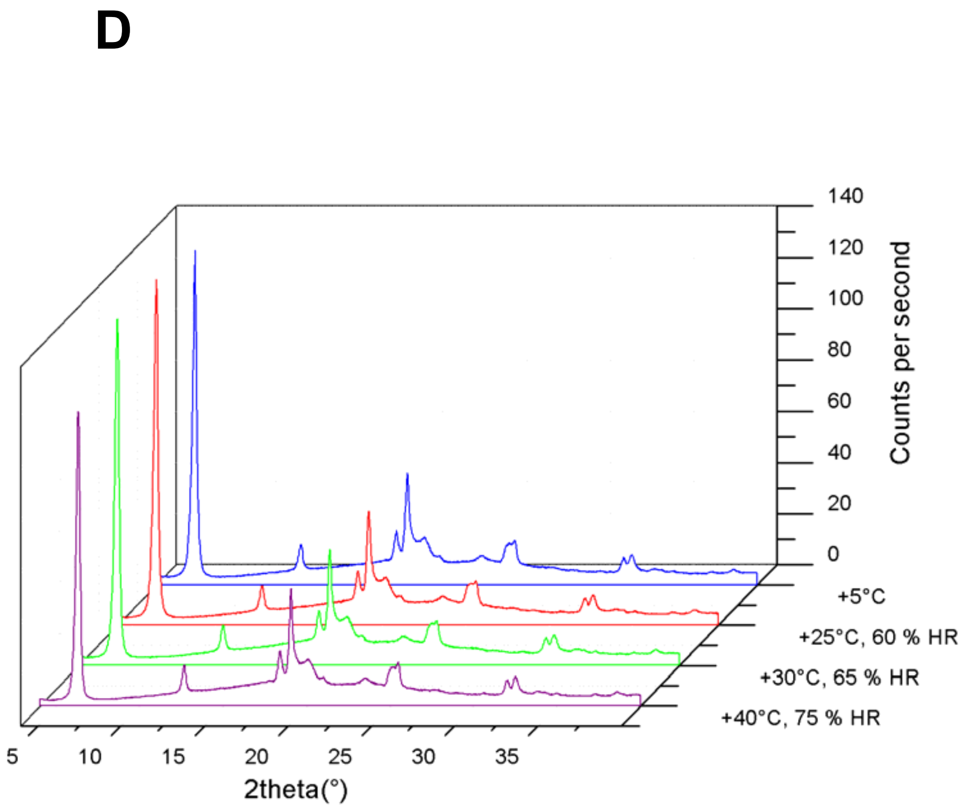

Figure S8. XRD profiles of optimized spray-dried powder containing LUZ19 stored at 5 °C (A) and 40 °C, 75% RH (B) over time, and at day 1 (C) and at 1 year (D) storage under different ICH conditions.

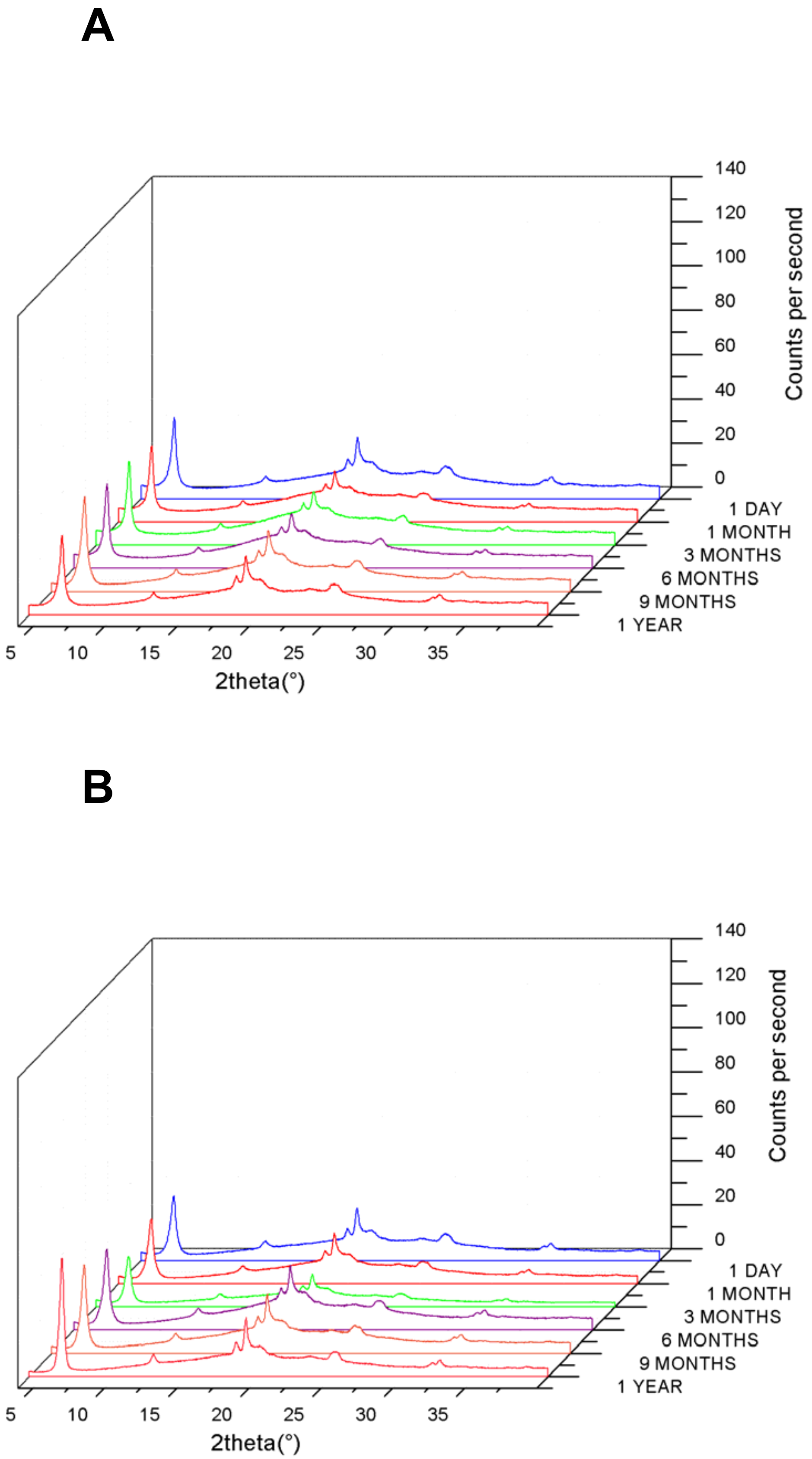
A
140
120
100
80
60
40
20
0
Counts per second
1 DAY
1 MONTH
3 MONTHS
6 MONTHS
9 MONTHS
1 YEAR
5
10
15
20
25
30
35
2theta(°)
B
140
120
100
80
60
40
20
0
Counts per second
1 DAY
1 MONTH
3 MONTHS
6 MONTHS
9 MONTHS
1 YEAR
5
10
15
20
25
30
35
2theta(°)

C

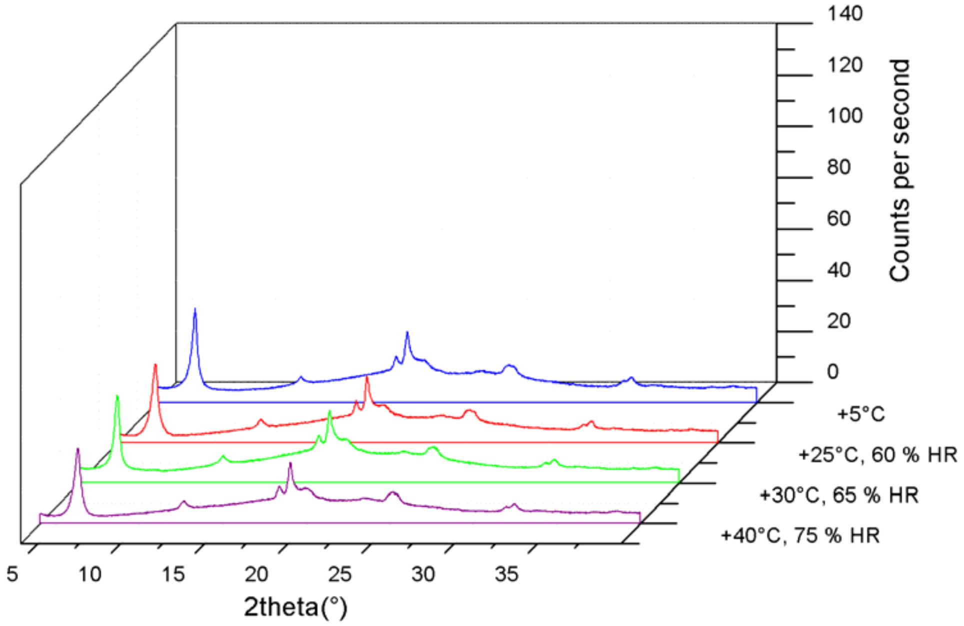

D

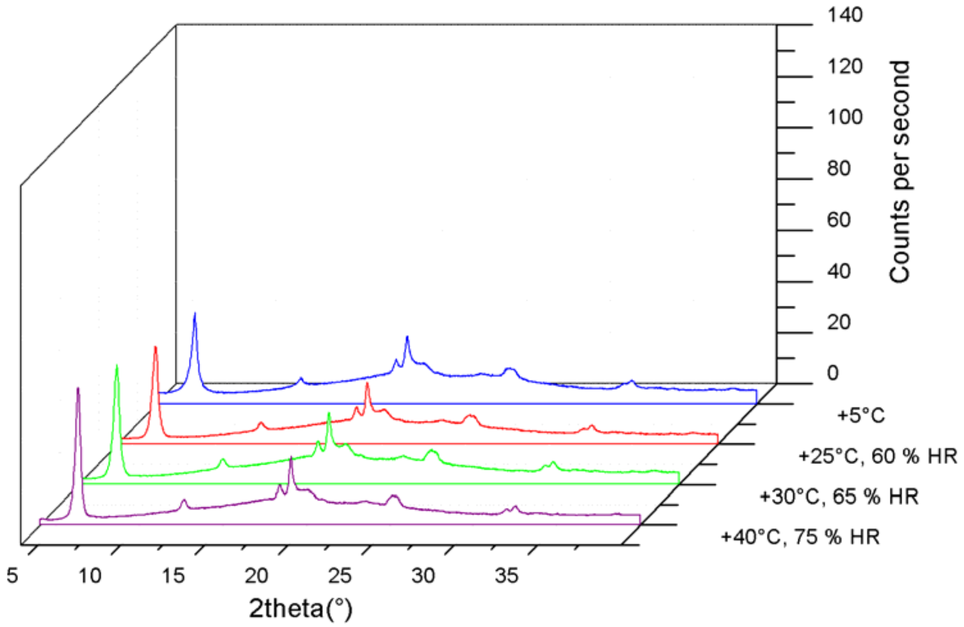

Figure S9. XRD profiles of optimized spray-dried powder containing 14-1 stored at 5 °C (A) and 40 °C, 75% RH (B) over time, and at day 1 (C) and at 1 year (D) of storage under different ICH conditions.
